# Interaction between Gelatin and Mulberry Leaf Polysaccharides in Miscible System: Physicochemical Characteristics and Rheological Behavior

**DOI:** 10.3390/foods11111571

**Published:** 2022-05-26

**Authors:** Xiu-Xiu Zhang, Bu-Yan Liao, Zi-Jing Guan, Kiran Thakur, Mohammad Rizwan Khan, Rosa Busquets, Jian-Guo Zhang, Zhao-Jun Wei

**Affiliations:** 1 School of Food Science and Biological Engineering, Hefei University of Technology, Hefei 230009, China; 2020111406@mail.hfut.edu.cn (X.-X.Z.); liaobuyan@afc.edu.cn (B.-Y.L.); guanzj@mail.hfut.edu.cn (Z.-J.G.); kumarikiran@hfut.edu.cn (K.T.); zhangjianguo@hfut.edu.cn (J.-G.Z.); 2 Ningxia Key Laboratory for the Development and Application of Microbial Resources in Extreme Environments, School of Biological Science and Engineering, North Minzu University, Yinchuan 750021, China; 3 Department of Chemistry, College of Science, King Saud University, Riyadh 11451, Saudi Arabia; mrkhan@ksu.edu.sa; 4 School of Life Sciences, Pharmacy and Chemistry, Kingston University London, Kingston upon Thames KT1 2EE, UK; r.busquets@kingston.ac.uk

**Keywords:** gelatin, mulberry leaf polysaccharides, miscible system, zeta potential, rheological properties

## Abstract

In this study, the miscible system was formed by mixing gelatin (G) with mulberry leaf polysaccharides (MLPs) continuously extracted with a hot buffer (HBSS), a chelating agent (CHSS), a dilute alkali (DASS), and a concentrated alkali (CASS), and the zeta potential, turbidity, particle size, distribution, and rheological properties of the miscible systems were evaluated. Under acidic conditions, the miscible systems of four polysaccharides and gelatin were in a clear state; under alkaline conditions, G-HBSS and G-CHSS were clarified, and G-DASS and G-CASS changed from clarification to turbidity. The zeta potential changed from positive to negative with the increase in pH. When the pH was at 7, it increased with the increase in polysaccharide concentration but was still negative. The four miscible systems all showed polydispersity. The particle sizes of G-HBSS and G-CHSS decreased with the increase in pH, while the particle sizes of G-DASS and G-CASS were increased. The four miscible systems showed “shear thinning” behavior, and the addition of gelatin reduced the apparent viscosity of the four polysaccharide solutions. G-CHSS was highly stable, and G-CASS was more suitable as a stabilizer in the freezing process.

## 1. Introduction

Mulberry, belonging to the mulberry genus of the mulberry family, is widely distributed throughout the world [1]. In particular, the planting area of mulberry trees in China ranks first in the world, and the yield of mulberry leaves is rich [2]. Mulberry leaves are mainly used for silkworm breeding, and a few are used for the preparation of tea and fruit juice [3,4]. In China, mulberry leaf is also a medicinal resource which is widely used in traditional Chinese medicine [5]. Mulberry leaves contain a variety of functional components, such as alkaloids, polyphenols and flavonoids, proteins, amino acids, and carbohydrates [6,7,8,9]. A variety of functional components endow mulberry leaves with different biological activities, such as hypoglycemic, anti-atherosclerotic, antioxidant, and antibacterial activities [10,11,12,13]. Though there are many studies on the active components of alkaloids, flavonoids, and polyphenols in mulberry leaves, in recent years, more studies have emerged on the activity of polysaccharides.

Plant polysaccharides have attracted great attention in recent years because of their various biological activities, such as antitumor, antiviral, hypoglycemic, and antioxidant activities [14,15,16,17]. Because of their rheological properties, polysaccharides can also be used as adhesives and gelling agents in the food and cosmetics industries [18]. Previous studies have shown that plant polysaccharides can be used as a source of natural antioxidants in the food, pharmaceutical, and other industries [19]. At the same time, because of their rheological properties, plant polysaccharides can be used as thickeners and adhesives in natural materials and food industries [20].

The food system is a complex system composed of compounds with different properties, such as polysaccharides, proteins, and minerals. The current research focus is on regulating and controlling the interaction of biological macromolecules in the process of food processing to change their characteristics or structure [21]. Polysaccharides and proteins are the main nutrients in food formulas and can be miscible under certain conditions and change the system by forming the electrostatic complex [22]. This change affects the food structure and rheological properties, which is of great significance in food processing. As a kind of protein macromolecule, gelatin has been widely used in food processing [23].

In our previous study, four mulberry leaf polysaccharides (MLPs) were extracted continuously with a thermal buffer, a chelating agent, dilute alkalis, and concentrated alkali extractant to obtain thermal buffer soluble solids (HBSSs), chelating agent soluble solids (CHSSs), dilute alkali soluble solids (DASSs), and concentrated alkali soluble solids (CASSs), respectively [19,20]. Four MLPs had a shear-thinning characteristic and the properties of non-Newtonian fluid [20], and exhibited significant differences in the physicochemical and antioxidant activities. The CASS displayed higher thermal properties and suitability as a supplement in hot processed foods [19]; The DASS showed the highest apparent viscosity at different tested concentrations [20]. However, there are no reports on the interaction between mulberry leaf polysaccharides and gelatin in the miscible system. In this study, the effects of gelatin on zeta potential, turbidity, particle size, distribution, and rheological properties of gelatin–polysaccharide miscible systems were studied.

## 2. Materials and Methods

### 2.1. Materials and Chemicals

The mulberry leaves (*Morus alba* L.) used in the experiment were provided by Anhui Academy of Agricultural Sciences (Anhui Province, Hefei, China). Four types of mulberry leaf polysaccharides (HBSS, CHSS, DASS, and CASS) were obtained by continuous extraction with four solvents [19,20]. All chemicals were analytical-grade.

### 2.2. Preparation of Gelatin–Polysaccharide Miscible Solution

A total of 0.5 g gelatin was dissolved in 100 mL water at 40 °C, 1 h, then stored at 4 °C for 24 h, and soaked in water at 25 °C for 18 h to prepare a stable gelatin solution with a mass concentration of 5 mg/mL [24]. Then, 0.1 g of mulberry leaf polysaccharides was added to 100 mL of 5 mg/mL gelatin solution and stirred until it was completely dissolved to obtain gelatin–polysaccharide miscible solutions (G-MLPs) with a concentration of 1 mg/mL for the phase diagram experiment, in which 2.3. 5 mg/mL gelatin solution was used as the solvent for the preparation of gelatin–polysaccharide solutions with different concentrations.

### 2.3. Phase Diagram

The gelatin–polysaccharide miscible solutions prepared in Section 2.2. were used as a mother liquor to prepare gelatin–polysaccharide miscible systems with different pHs (3–10, interval 0.5). At first, the miscible system was placed at room temperature for 1 h, and then the solution state was observed. The state was divided into the following three types: clear solution, cloudy solution, precipitation, and cloudy solution [25]. The phase diagram was drawn according to the change in pH.

### 2.4. Measurement of Zeta Potential, Particle Size, and Distribution

The zeta potential, particle size, and distribution of gelatin–polysaccharide miscible solution at different pHs (3.5, 7, 10) with a concentration of 20 mg/mL and different concentrations (5, 10, 15, and 20 mg/mL) at pH 7 were determined by Malvin particle size analyzer (Zetasizer Nano-ZSE, Malvern Instruments, Worcestershire, UK) [26]. The calculations of zeta potential and particle size were as follows:

(1) $$\zeta \left(\times 10^{3}\right)=\frac{3{\;\eta \;U}_{E}}{2_{\varepsilon f}\;\left(k\alpha \right)}$$

ε (F/M) is numerically equal to εrε0; εr is the relative permittivity of the mixture (25 °C water is about 78.2); ε0 is the vacuum relative permittivity (about 8.854 × 10^−12^ F/m); η (Pa·s) is the viscosity of the dispersion system (25 °C water is about 8.937 × 10^−4^ Pa·s); f (κα) is the ratio of the particle radius α to Debye’s length in Henry’s equation and approximately 1.5 in the Smoluchowski model.

(2) $$d\left(\times 10^{9}\right)=\frac{K_{B}T}{3\pi \;\eta \;D}$$

η (Pa·s) is the viscosity of the dispersion system (25 °C water is about 8.937 × 10^−4^ Pa·s); KB is Boltzmann’s constant, and T is absolute temperature (k); the refractive index was set as follows: water 1.33, G 1.45, G-MLPs 1.59; the absorbance of the material was 0.01.

### 2.5. Determination of Rheological Properties

#### 2.5.1. Effect of Concentration, pH, Na^+^ Concentration, and Temperature on Apparent Viscosity of G-MLPs

The rheological properties of G-MLPs were determined using the previously described method [20]. The rheological properties of the gelatin–polysaccharide miscible system were mainly studied through the effects of concentration, pH, Na^+^ concentration, and temperature on the apparent viscosity of G-MLPs. In short, gelatin–polysaccharide aqueous solutions with concentration gradients of 5, 10, 15, and 20 mg/mL were prepared to study the effect of concentration on apparent viscosity, and 5 mg/mL gelatin solution was used as control. The pH value of 10 mg/mL gelatin–polysaccharide aqueous solution was adjusted to 3.5, 7.0, and 10.0 with HCl and NaOH solution to study the effect of pH on apparent viscosity. NaCl solutions with concentrations of 0, 0.1, 0.2, 0.4, and 0.8 mol/L were prepared, respectively, and then gelatin was dissolved and prepared in 5 mg/mL gelatin–NaCl solution, and finally polysaccharides were added to the solution to make the concentration of polysaccharides 10 mg/mL. When studying the effect of temperature on the apparent viscosity of the miscible system, we selected −20, 25, and 100 °C for cold and heat treatment of 10 mg/mL gelatin–polysaccharide miscible systems. Rheometer (DHR-3, TA instruments, New Castle, DE, USA) conditions were as follows: steady-state shear mode; clamp: diameter 40 mm; cone plate: cone angle 2°; shear speed range: 0.01–1000 s^−1^; temperature: 20 °C.

#### 2.5.2. Effect of G-MLPs on Viscoelasticity

The changes in apparent viscosity of gelatin–polysaccharide aqueous solution with mass concentrations of 5, 10, 15, and 20 mg/mL were measured under different shear oscillation frequencies. Test conditions were as follows: DHR-3: clamp: diameter 40 mm; cone plate: cone angle 2°; shear speed range: 0.01–100 Hz; temperature: 20 °C [20].

### 2.6. Statistical Analysis

All the tests reported were repeated three times. One-way ANOVA through SPSS 21.0 was used for the data analysis at *p* < 0.05, and origin 8.0 software was used for graphs.

## 3. Results and Discussion

### 3.1. Phase Diagram of Gelatin–Mulberry Leaf Polysaccharides (G-MLPs) Miscible System

Table 1 shows the effect of pH on the phase behavior of the gelatin–mulberry leaf polysaccharide miscible system. The electrical properties of proteins were reported to vary according to the pH of their environment. When it was in an electronegative state, proteins co-dissolved with anionic polysaccharides due to electrostatic repulsion, and under electropositive conditions, proteins aggregated due to the electrostatic binding reaction with anionic polysaccharides, resulting in a cloudy and precipitated solution [27]. At pH < 7, the dispersions of the four miscible systems were clear. When pH was 7–8.5, G-HBSS and G-CHSS were still clear, indicating that the reaction between G-HBSS and G-CHSS affected the intermolecular aggregation of polysaccharides [22]. G-DASS and G-CASS were the alkaline extracts; therefore, their resulting systems changed from clear to cloudy. When the pH was close to alkaline conditions, the repulsion force between G-DASS and G-CASS molecules decreased, resulting in aggregation, and the miscible system changed from clear to cloudy [25]. With the continuous increase in pH value, both the G-HBSS and G-CHSS miscible systems changed from clear to cloudy, while G-DASS and G-CASS changed to cloudy and precipitated systems. At pH 9, the four samples were unstable, indicating that the system environment with high pH was conducive to the formation of an insoluble gelatin–polysaccharide complex, which ultimately increased the instability of the system [25].

### 3.2. Zeta Potential Analysis of Gelatin–Mulberry Leaf Polysaccharides (G-MLPs) Miscible System

**Figure 1:**
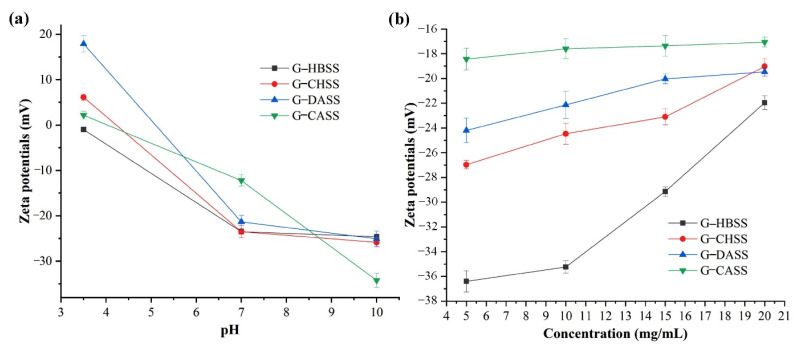
Zeta potential of G-MLPs at 20 mg/mL as a function of pH (**a**); zeta potential of G-MLPs as a function of concentration at pH 7 (**b**).

The change in the zeta potential of gelatin and mulberry leaf polysaccharide miscible systems with respect to pH is shown in Figure 1a. With the increase in pH value, the zeta potential of the four miscible systems G-HBSS, G-CHSS, G-DASS, and G-CASS changed from positive to negative. Moreover, the zeta potential of the miscible system also decreased with the increase in pH. The zeta potential of the miscible system was negatively charged with the increase in pH, which may have been due to the electrostatic binding reaction between gelatin and mulberry polysaccharides, and the deprotonation reaction of amino and carboxyl functional groups on gelatin molecules and carboxyl groups in the mulberry polysaccharide structure with the increase in pH [28]. At pH 7.0, with the increase in mulberry leaf polysaccharide concentration, the zeta potential of the miscible system gradually increased, but it was still negative, as shown in Figure 1b. This may be due to the fact that the combination of mulberry leaf polysaccharides and gelatin resulted in the movement of the net charge of the miscible system to the positive direction [29,30].

### 3.3. Particle Size Analysis of Gelatin–Mulberry Leaf Polysaccharides (G-MLPs) Miscible System

**Figure 2:**
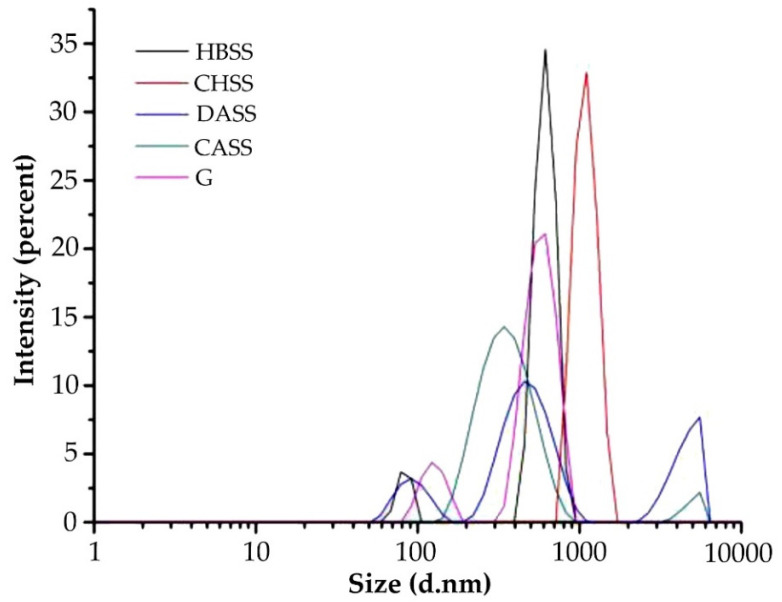
Particle size distribution of MLPs and gelatin (G).

The particle size and distribution of the gelatin solution and four mulberry leaf polysaccharide solutions at the same concentration (10 mg/mL) are shown in Figure 2. The particle size of the HBSS, CHSS, DASS, and CASS had two, one, three, and two peaks, respectively. Unlike the DASS, the other three mulberry leaf polysaccharides were monodisperse, indicating that the composition of the HBSS, CHSS, and CASS was relatively uniform and may have formed electrostatic complexes with the aqueous solution. This result is consistent with the particle size distribution of water-soluble polysaccharides extracted from kidney beans [31]. The particle size of gelatin had two peaks, and the particle size was larger, which may have been due to the high pH value of the solution, which led to its positive or negative charge and increased the interaction between gelatin and water molecules [32].

**Figure 3:**
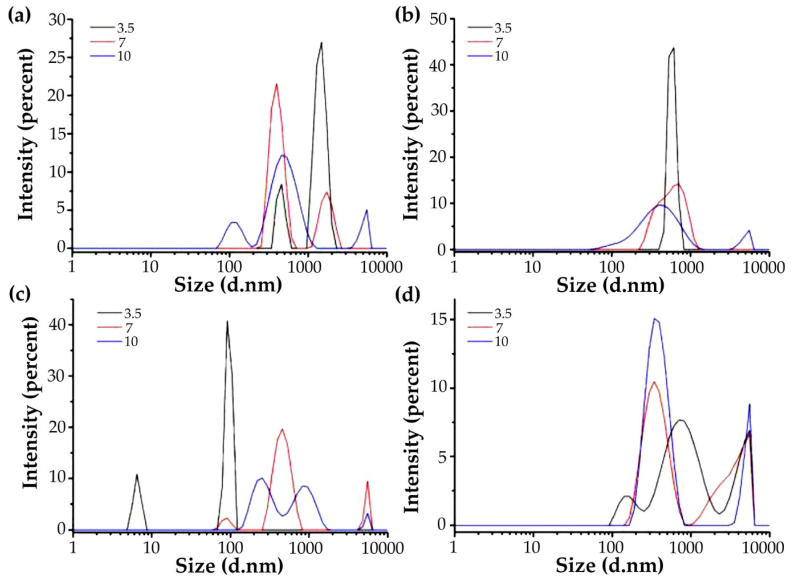
Particle size distribution of G-MLPs at different pHs. (**a**) G-HBSS; (**b**) G-CHSS; (**c**) G-DASS; (**d**) G-CASS.

Figure 3 shows the particle size and distribution of four miscible systems at pH 3.5, 7.0, and 10.0. At pH 3.5, G-HBSS, G-CHSS, G-DASS, and G-CASS formed two, one, two, and three main peaks, respectively. When the pH was 7, there were two, one, three, and two peaks, respectively. When the pH was 10, there were three, two, three, and two peaks, respectively. Compared with the particle size of gelatin and four polysaccharides, the particle size distribution of the four miscible systems of gelatin and mulberry leaf polysaccharides changed significantly. Under different pH conditions, the G-CHSS solution still maintained high monodispersity, which may have been caused by the electrostatic complex formed after the miscibility of the CHSS and gelatin [30]. The three miscible systems of G-HBSS, G-DASS, and G-CASS showed polydispersity, which may have been due to the charge repulsion between gelatin and these three mulberry leaf polysaccharides [33]. With the increase in pH, the change trend of particle size of the four miscible systems was also different. The particle size of G-HBSS and G-CHSS miscible systems decreased with the increase in pH, while the particle size of G-DASS and G-CASS increased with the increase in pH. These phenomena may have been caused by the different extractants of the HBSS, CHSS, DASS, and CASS. Specifically, the reason for the change trend of G-HBSS and G-CHSS may be that with the increase in pH, gelatin and two polysaccharides produced repulsion, which reduced the particle size of the complex [30], while the reason for the change of G-DASS and G-CASS may be that both were alkaline extracts. Under alkaline conditions, the electrostatic complexation reaction intensity between gelatin and polysaccharides increased, and the formed complex was easy to aggregate, resulting in the increase in particle size, which was the same as the change of the solution state reflected in the above phase diagram [33].

**Figure 4:**
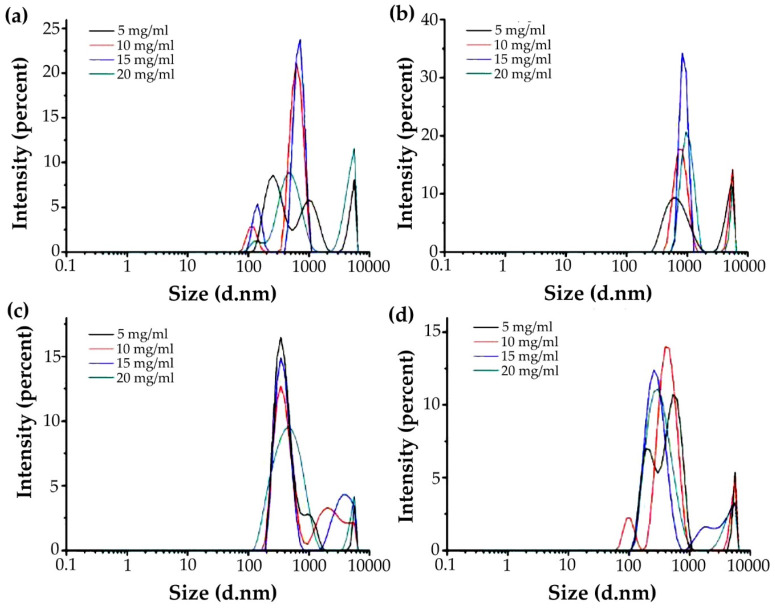
Particle size distribution of G-MLPs at different concentrations. (**a**) G-HBSS; (**b**) G-CHSS; (**c**) G-DASS; (**d**) G-CASS.

The particle size distribution and size of the four miscible systems at different concentrations are shown in Figure 4. It can be seen from the figure that the miscible system showed polydispersity, but the particle size did not change much with the change in concentration, indicating that the change in concentration only affected the distribution of particle size, and had little effect on its size. This may be because with the increase in the polysaccharide concentration, it had little effect on the intermolecular force of the miscible system of gelatin and polysaccharides [32].

### 3.4. Rheological Property of Gelatin–Mulberry Leaf Polysaccharide (G-MLPs) Miscible System

#### 3.4.1. Effect of Concentration on Apparent Viscosity of G-MLPs

**Figure 5:**
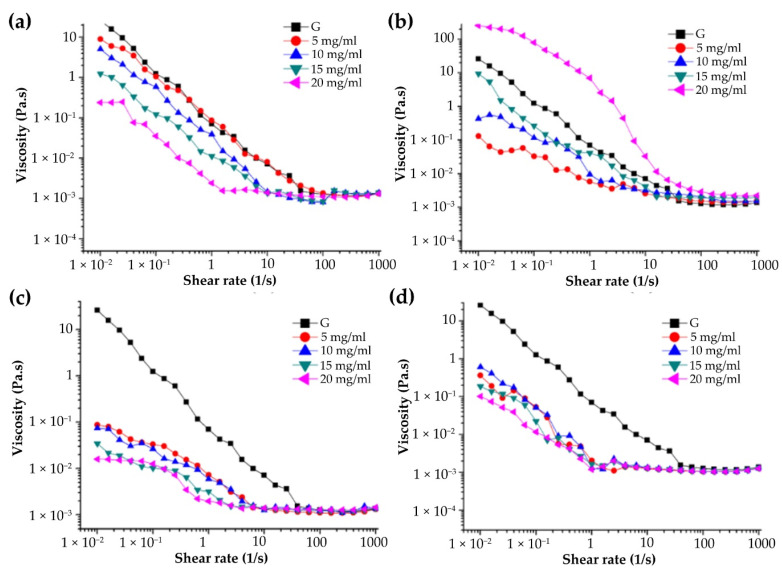
Effect of different concentrations on the viscosity of G-MLPs. (**a**) G-HBSS; (**b**) G-CHSS; (**c**) G-DASS; (**d**) G-CASS.

The change in and influence of apparent viscosity of four miscible systems with different concentrations and shear rates and the comparison with the apparent viscosity of the gelatin solution are shown in Figure 5. Under the condition of a low shear rate, the viscosity of the four miscible systems decreased with the increase in shear rate, and they showed the “shear thinning” behavior [20]. The main reason for the “shear thinning” behavior was that gelatin and polysaccharide were colloidal particles of macromolecules. Under the condition of a static or low shear rate, they entangled with each other and had a higher viscosity, while with the increase in shear rate, the shear stress between the flow layers increased, which made the scattered gelatin–polysaccharide chain particles roll, rotate, and shrink into clusters, resulting in reducing the hooking effect between the chain molecules and the viscosity of gelatin–polysaccharide solutions. On the other hand, with the increase in flow rate, the molecular force between miscible systems decreased, the flow direction of gelatin–polysaccharide macromolecules changed from disorder to ordered, the flow direction appeared consistent, and the viscosity decreased [34]. This was a typical non-Newtonian fluid behavior, indicating that the miscible systems were a non-Newtonian fluid like pure polysaccharide solutions. As mentioned above, “Shear thinning” behavior is important in food processing industries for the development of various food products with desirable properties [20].

The apparent viscosity of the four miscible systems varied with the increase in mass concentration (Figure 5). Except for the apparent viscosity of the G-CHSS miscible system at the highest concentration, which was higher than that of the gelatin solution, the apparent viscosity of other miscible systems was lower than that of the gelatin solution, which indicated that the addition of polysaccharides reduced the apparent viscosity of the gelatin solution. Specifically, after the four mulberry leaf polysaccharide solutions were mixed with the gelatin solution, the apparent viscosity of G-HBSS, G-DASS, and G-CASS miscible systems decreased with the increase in mass concentration, while the apparent viscosity of the G-CHSS miscible system increased with the increase in mass concentration, which showed relatively appreciable thickening behavior of G-CHSS. One study reported that the apparent viscosity of polysaccharides increased with their concentration, while their apparent viscosity decreased after the addition of gelatin, which may be related to the fact that the particle size of the miscible system in the particle size experiment did not increase with the increase in solution concentration [35]. The previous studies [18,20] showed that the apparent viscosity increased with the increase in mass concentration, mainly because the fluid viscosity results from intermolecular internal friction. In this study, the particle size in the miscible system did not increase, resulting in the decrease in apparent viscosity. Within the studied concentration range, the fluid types of gelatin and mulberry leaf polysaccharide miscible systems did not change due to the change in concentration.

It can also be seen from Figure 5 that the rheological properties of the solutions of the miscible systems with different concentrations were the same as those of mulberry leaf polysaccharides. With the increase in concentration, the pseudoplastic flow was dominant. When the shear rate was 0.01–10 s^−1^, the viscosity of the four miscible systems of G-HBSS, G-CHSS, G-DASS, and G-CASS decreased rapidly with the increase in shear rate, and then became stable as the shear rate increased. With the increase in shear rate, the miscible system showed the properties of Newtonian fluid, which is characterized by the gradual stabilization of apparent viscosity. Compared with pure mulberry leaf polysaccharides, G-CHSS had the highest apparent viscosity, and G-DASS had the lowest apparent viscosity among the four miscible systems. The above results show that G-CHSS can be used as a food thickener, gelling agent, and binder in food, medicine, and cosmetics industries [18].

#### 3.4.2. Effect of pH on Apparent Viscosity of G-MLPs

**Figure 6:**
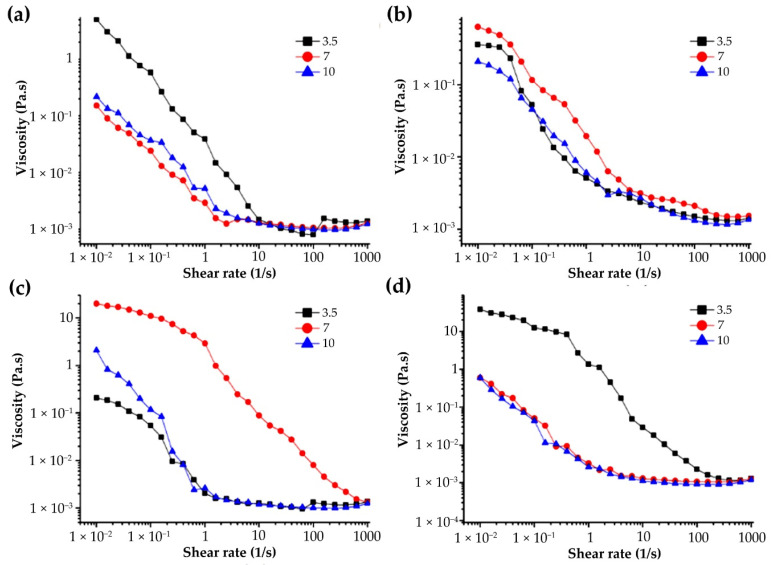
Effect of pH on the viscosity of G-MLPs. (**a**) G-HBSS; (**b**) G-CHSS; (**c**) G-DASS; (**d**) G-CASS.

The four miscible systems showed different apparent viscosity changes in low- and high-pH solutions (Figure 6). G-CHSS and G-DASS showed a decrease in apparent viscosity under acidic and alkaline conditions, which may be because the intermolecular force of the miscible system was weakened in the acidic or alkaline environment, resulting in the decomposition of the polymer of the polysaccharide and gelatin miscible system and a decrease in molecular weight and apparent viscosity [36]. G-HBSS and G-CASS had the highest apparent viscosity under acidic conditions, and the apparent viscosity decreased with the increase in pH. The reason for this may be that the two polysaccharide miscible systems belonged to an acidic polysaccharide miscible system, and their structure was much more stable under acidic conditions, while in the alkaline environment, the solvation effect of water increased with the increase in OH- in the solution, which may have broken the intramolecular and intermolecular hydrogen bonds of the molecules and reduced the interactions, resulting in making the structure of the miscible system loosen and decreasing the viscosity of G-MLPs [37]. Among the four miscible systems, the CHSS had the highest stability, and it can be used as an important ingredient in acidic or alkaline beverages and other products.

#### 3.4.3. Effects of Na^+^ Concentration on Apparent Viscosity of G-MLPs

**Figure 7:**
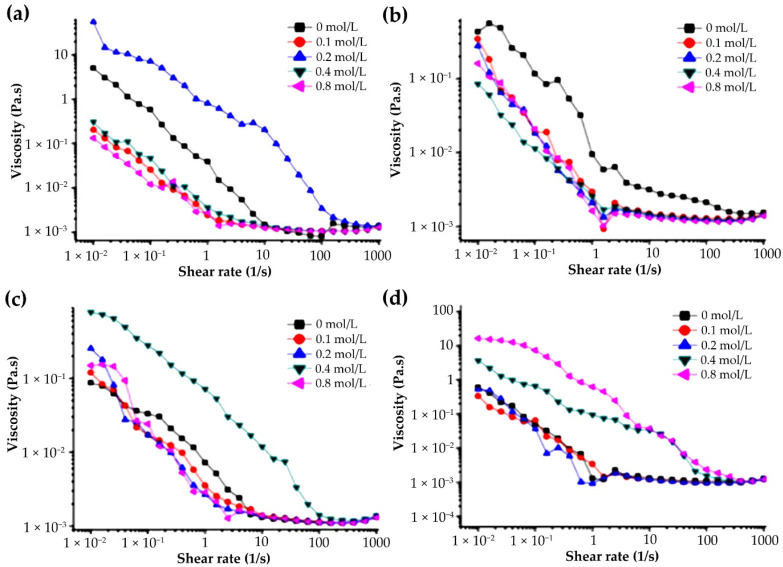
Effect of Na^+^ on the viscosity of G-MLPs (10 mg/mL). (**a**) G-HBSS; (**b**) G-CHSS; (**c**) G-DASS; (**d**) G-CASS.

Figure 7 shows the effect of Na^+^ concentration on the apparent viscosity of 10 mg/mL gelatin–polysaccharide miscible systems. It can be seen from the figure that the apparent viscosity of G-CASS (Figure 7b) increased with the increase in Na^+^ concentration. The reason for this phenomenon may be that the addition of Na^+^ affected the molecular conformation of the miscible system and improved the structural entanglement, resulting in the increase in apparent viscosity [38,39]. The apparent viscosity of G-CHSS decreased with the increase in Na^+^ concentration (Figure 7b). This may be due to the salting out of the polysaccharide solution of the miscible system due to the addition of Na^+^, which would reduce the concentration of the polysaccharide solution and the apparent viscosity. The higher the NaCl concentration, the more serious the salting out phenomenon and the greater the viscosity drop [18,20]. The apparent viscosity of G-HBSS and G-DASS did not change regularly with the concentration of Na^+^, which may have been caused by the particularity of the structure of the HBSS and DASS. The action law of Na^+^ on their miscible systems was different from that of G-CHSS and G-CASS. In other words, the low concentration of Na^+^ increased the structural entanglement between molecules, resulting in the increase in apparent viscosity, while the high concentration of Na^+^ salted out the system, resulting in the decrease in apparent viscosity [18]. The inconsistency of the rheological behavior of samples may also have been due to the different intensity of the interaction between gelatin and the HBSS, CHSS, DASS, and CASS in different concentrations of sodium chloride solution. Therefore, during the food processing, the content of salt ions needs to be strictly controlled to ensure the desired products.

#### 3.4.4. Effect of System Temperature on Apparent Viscosity of G-MLPs

**Figure 8:**
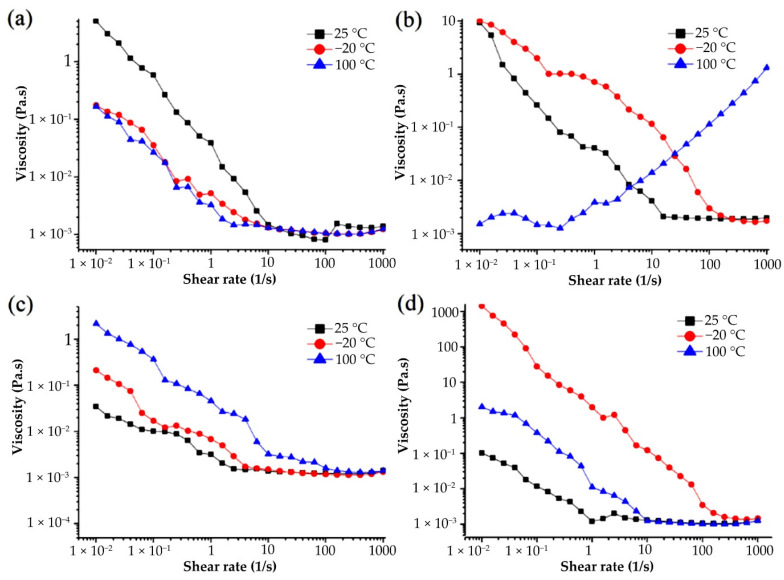
Effect of freezing and heating on the viscosity of G-MLPs. (**a**) G-HBSS; (**b**) G-CHSS; (**c**) G-DASS; (**d**) G-CASS.

The different effects of cold treatment and heat treatment on the apparent viscosity of the four miscible systems are shown in Figure 8. Gelatin is thermo-reversible, meaning that the viscosity of a gelatin solution would increase with decreasing temperature to form a gel, and vice versa. Thus, heating and freezing followed by reversing the system temperature to room temperature will have no significant effect on gelatin viscosity [39]. The rheological properties of the four miscible systems after cold and heat treatment were different. After cold treatment, the apparent viscosity of G-HBSS decreased, while the apparent viscosity of G-CHSS, G-DASS, and G-CASS was increased. After heat treatment, the apparent viscosity of G-HBSS was decreased. The increase in the apparent viscosity of G-DASS was higher than that of cold treatment, while the increase in the apparent viscosity of G-CASS was lower than that of cold treatment, and the apparent viscosity of G-CASS after freezing treatment was the highest among the four miscible systems, which indicated that G-CASS was more suitable as a stabilizer in the freezing process. After heat treatment, the apparent viscosity of the G-CHSS sample generally showed an upward trend with the increase in shear rate and changed from “shear thinning” to “shear thickening” expansive fluid [40]. The reason for this phenomenon may be that the interaction between gelatin and the CHSS was strengthened during the heat treatment, resulting in the very stable miscible system. When the temperature returned to room temperature, it was still in an orderly distribution. Therefore, with the increase in shear rate, the order in the system was disturbed and transformed into disorder, and the apparent viscosity increased with the increase in shear force. The above results show that the four miscible systems, except G-HBSS, had good freeze–thaw stability and could be applied to the products that need to be stabilized after freezing, while the “shear thickening” characteristic of G-CHSS marks its suitability as a thickener in heat processing.

#### 3.4.5. Effect of G-MLPs on Viscoelasticity

**Figure 9:**
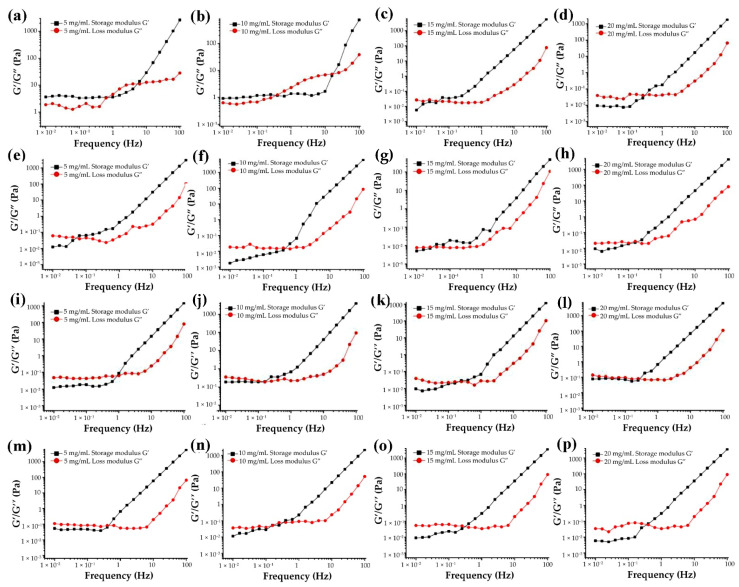
Frequency dependence of storage (G’) and loss (G”) modulus of G-HBSS (**a**–**d**), G-CHSS (**e**–**h**), G-DASS (**i**–**l**), and G-CASS (**m**–**p**) at different concentrations.

At 25 °C, the storage modulus G’ and loss modulus G” of gelatin and mulberry leaf polysaccharide miscible systems changed with frequency, as shown in Figure 9. By dynamically measuring the G’ and G” of the samples, the advantages of solid elasticity or liquid viscosity of the sample could be quantified [41]. Within the range of shear oscillation frequency measured in this study, the storage modulus G’ and loss modulus G” of the four miscible systems had a certain dependence on the shear oscillation frequency, which showed that they continued to increase with the increase in shear oscillation frequency and indicated that gelatin and mulberry leaf polysaccharides solutions were a viscoelastic material [18,20]. The storage modulus G’ and loss modulus G” of G-HBSS were different from those of the other three miscible systems. At 5 mg/mL and 10 mg/mL, G’ was greater than G” under the low shear vibration frequency of G-HBSS, and solution mainly showed the elastic properties of a solid. With the increase in frequency, G’ was less than G”, and the main property of the solution changed to the viscosity of the liquid. The frequency of shear oscillation further increased until the G’ of the solution was greater than G”. The elastic properties of the solid were dominant [42]. The changes between the storage modulus G’ and loss modulus G” of G-HBSS at 15 mg/mL and 20 mg/mL were the same as those of the other three miscible systems. At low frequency, the storage modulus G’ was less than the loss modulus G”, and the solution showed the viscosity of the liquid. With the increase in frequency, a crossover value is reported [43]. At this time, the loss modulus G’ curve and the storage modulus G” curve was close to or intersected, which also meant that the crossover value was negatively correlated with the elastic contribution at the beginning of the elastic behavior [44]. With the further increase in oscillation frequency, G’ began to exceed G”, which indicated that the elastic properties were dominant. Additionally, the miscible system mainly showed the elastic properties of solids. The crossover values of four gelatin and mulberry leaf polysaccharide miscible systems varied with different concentrations. At 5 mg/mL and 15 mg/mL, the crossover value of G-CHSS was lower than that of the other three, while at 10 mg/mL and 20 mg/mL, the crossover value of G-DASS was lowest.

## 4. Conclusions

The zeta potential, turbidity, rheological properties, and particle size of gelatin–mulberry leaf polysaccharide miscible systems were evaluated. Under acidic conditions, the four miscible systems were in a clear state. Under alkaline conditions, G-HBSS and G-CHSS were in a clear state, while G-DASS and G-CASS changed from a clear to cloudy state. With the increase in pH, the potential of the four miscible systems changed from positive to negative and decreased gradually. At pH 7.0, the zeta potential of the four miscible systems increased with the increase in MLP concentration, but it was still negative. The particle size distribution of the four miscible systems showed polydispersity at different concentrations, but the particle size did not change much. The four miscible systems showed “shear thinning” behavior, and the addition of gelatin reduced the apparent viscosity of four polysaccharides solutions. The apparent viscosity of G-HBSS, G-DASS, and G-CASS decreased with the increase in mass concentration, while the apparent viscosity of the G-CHSS miscible system increased with the increase in mass concentration, and it had relatively appreciable thickening. The effect of pH value on the stability of the miscible system was in the order of G-CHSS > G-HBSS > G-CASS > G-DASS. Na^+^ had different effects on the apparent viscosity of the solutions of the four miscible systems. With the increase in Na^+^ concentration, the apparent viscosity of G-CASS increased, while G-HBSS, G-CHSS, and G-DASS decreased. After cold and heat treatment, G-CHSS changed from a “shear thinning” to “shear thickening” expansive fluid. Dynamic oscillatory shear data revealed that the miscible systems exhibited viscous properties with the increase in oscillation frequency.

## Figures and Tables

**Table 1:** State diagrams of G-MLPs’ mixed solutions (1 mg/mL) as a function of pH.

Samples	pH													
	3	3.5	4	4.5	5	5.5	6	7	7.5	8	8.5	9	9.5	10
G-HBSS	▲	▲	▲	▲	▲	▲	▲	▲	▲	▲	▲	■	■	■
G-CHSS	▲	▲	▲	▲	▲	▲	▲	▲	▲	▲	▲	■	■	■
G-DASS	▲	▲	▲	▲	▲	▲	▲	▲	■	■	■	●	●	●
G-CASS	▲	▲	▲	▲	▲	▲	▲	▲	■	■	■	●	●	●

Note: The solubility or insolubility was evaluated by visual observation. (▲: clear solution; ■: cloudy solution; ●: precipitation and cloudy solution).

## Data Availability

Not applicable.
